# Visual prognosis of Behçet’s uveitis patients in China and its associated factors

**DOI:** 10.3389/fimmu.2026.1617653

**Published:** 2026-03-17

**Authors:** Minghang Pei, Yujing Qian, Xinshu Liu, Chan Zhao, Fei Gao, Meifen Zhang

**Affiliations:** 1 Department of Ophthalmology, The First Affiliated Hospital of Zhengzhou University, Zhengzhou, China; 2 Department of Ophthalmology, Peking Union Medical College Hospital, Chinese Academy of Medical Sciences and Peking Union Medical College, Beijing, China; 3 Department of Ophthalmology, The Fourth People’s Hospital of Shenyang, Shenyang, China

**Keywords:** Behçet's uveitis, visual prognosis, Chinese population, macular damage, optic nerve atrophy, biologic therapy

## Abstract

**Background:**

Behçet’s disease (BD) is a chronic, relapsing, multisystem inflammatory disorder, with ocular involvement being a major cause of irreversible visual impairment. Behçet’s uveitis (BU) often presents as bilateral, recurrent, non-granulomatous panuveitis with occlusive retinal vasculitis. Despite advances in immunosuppressive and biologic therapies, long-term visual outcomes remain variable, and factors associated with poor prognosis, particularly in Chinese populations, are not well defined.

**Methods:**

We conducted a retrospective cohort study of 153 Chinese patients (268 eyes) with BU at Peking Union Medical College Hospital from February 2003 to February 2023. Clinical records, ocular examinations, and systemic manifestations were reviewed. The primary endpoint was severe visual impairment (best-corrected visual acuity [BCVA] <20/200) at the final follow-up. Secondary endpoints included ocular complications and their impact on visual outcomes. Kaplan–Meier survival analysis and mixed-effects logistic regression (accounting for inter-eye correlation) were used to evaluate risk factors for poor visual prognosis.

**Results:**

Among the cohort, 77.8% were male, and bilateral involvement occurred in 84.3%. Median follow-up was 30 months. Panuveitis was the predominant form (90.3%), and cataract (64.9%) and macular edema (63.1%) were the most common complications. At final follow-up, 25.4% of eyes had BCVA <20/200. Kaplan–Meier analysis showed cumulative risk of severe visual loss of 3.5% at 1 year, 11.0% at 2 years, 19.2% at 5 years, and 41.4% at 10 years. Male patients experienced visual impairment earlier than females. Mixed-effects logistic regression identified macular damage (p=0.027, OR = 3.70) and optic nerve atrophy (p<0.001, OR = 6.47) as significant predictors of poor visual outcomes, while complete-type BD showed a trend toward association (p=0.072, OR = 2.00). Systemic disease severity was not significantly associated with visual prognosis.

**Conclusion:**

In this Chinese cohort with BU, irreversible ocular structural damage, particularly macular damage and optic nerve atrophy, was the primary determinant of long-term visual outcomes. Systemic disease severity had limited predictive value. Despite improvements compared with historical data, these findings highlight the critical importance of early recognition and effective management of posterior segment inflammation to prevent permanent visual impairment.

## 1 Introduction

Behçet’s disease (BD) is a chronic, relapsing, multisystem inflammatory disorder characterized by recurrent oral aphthae, genital ulcers, and ocular inflammation (1, 2). As a variable-vessel vasculitis, BD may involve multiple organs, including the skin, joints, nervous system, gastrointestinal tract, and large vessels, leading to substantial morbidity over the disease course (3). Among these manifestations, ocular involvement remains one of the most serious complications and is a major cause of irreversible visual impairment in affected patients (2, 4).

Behçet’s uveitis (BU) typically presents as bilateral, recurrent, non-granulomatous panuveitis with occlusive retinal vasculitis (5). Despite advances in immunosuppressive and biologic therapies, BU continues to pose a significant threat to vision, particularly in patients with severe posterior segment involvement (6, 7). Visual outcomes vary widely among individuals, reflecting the marked heterogeneity of disease phenotype, inflammatory burden, and treatment response (8). Importantly, ocular involvement often occurs in young, working-age adults, and visual loss can severely affect quality of life, daily functioning, and socioeconomic productivity, making the long-term consequences of BU particularly burdensome (9).

Previous studies have identified several clinical and demographic factors associated with poorer visual prognosis in BU. Male sex has consistently been reported as a risk factor for more severe ocular disease, higher frequency of posterior or panuveitis, and worse long-term visual outcomes (10). Large registry-based and multicenter studies further suggest that male patients tend to accumulate greater irreversible organ damage, including ocular damage, compared with female patients. Early disease onset has also been linked to a more aggressive disease course, possibly due to prolonged inflammatory exposure over time (11). In addition, longer disease duration has been associated with cumulative structural damage of the retina and optic nerve, ultimately contributing to visual loss (12).

Specific ocular characteristics play a crucial role in determining prognosis. The presence of panuveitis, retinal vasculitis, and recurrent posterior segment inflammation has been repeatedly associated with poor visual outcomes (2). Genetic susceptibility factors, particularly human leukocyte antigen B51 (HLA-B51) positivity, have been linked to increased disease severity and a higher likelihood of ocular involvement, although their direct association with visual prognosis remains controversial (13).

Geographic and ethnic differences further influence the clinical expression and outcomes of BU (14). Most existing prognostic data originate from endemic regions along the ancient Silk Road, including Turkey, the Middle East, and parts of the Mediterranean basin (15). However, accumulating evidence indicates that disease phenotype, treatment patterns, and long-term outcomes may differ substantially across populations. In China, BD represents a distinct clinical entity with unique demographic characteristics, yet studies focusing specifically on visual prognosis and its associated factors remain limited.

Therefore, a comprehensive evaluation of visual outcomes in Chinese patients with BU is needed to better understand prognostic patterns within this population and to identify factors associated with irreversible visual impairment in real-world clinical practice. In this study, we aimed to investigate the long-term visual prognosis of Behçet’s uveitis patients in China and to analyze demographic, systemic, and ocular factors associated with poor visual outcomes.

## 2 Materials and methods

### 2.1 Study aims and endpoints

This retrospective study aimed to investigate the visual prognosis of Behçet’s uveitis (BU) in a Chinese cohort and to identify associated risk factors for visual impairment. The primary endpoint was the occurrence of severe visual impairment, defined as best-corrected visual acuity (BCVA) worse than 20/200 at the final follow-up. Secondary endpoints included the development of ocular complications (e.g., cataract, macular damage, retinal vascular occlusion, optic atrophy) and their impact on visual outcomes.

### 2.2 Study population and diagnostic criteria

The study was conducted in accordance with the Declaration of Helsinki and approved by the Institutional Review Board of Peking Union Medical College Hospital (PUMCH, IRB number: I-25PJO662). We reviewed medical records of patients diagnosed with Behçet’s disease (BD) who attended the Department of Ophthalmology at PUMCH between February 2003 and February 2023.

Diagnosis of BD was established according to the SUN (Standardization of Uveitis Nomenclature) classification criteria, which require fulfillment of the International Study Group for BD criteria (16). Patients were included if they met the following criteria (1) diagnosis of BD as defined above; (2) ocular involvement in at least one eye, presenting as uveitis; (3) availability of complete medical records; and (4) a minimum follow-up of 6 months with essential data for outcome evaluation.

Exclusion criteria comprised coexisting ocular conditions that could confound the assessment of BU, such as ocular trauma, diabetic retinopathy, age-related macular degeneration, other immune-mediated disorders, or syphilis infection. Patients or eyes with incomplete fundus imaging or missing critical data (e.g., visual acuity, disease onset time) were also excluded.

### 2.3 Assessment of uveitis activity and ocular evaluation

Uveitis activity was assessed using standardized ophthalmological examinations, including slit-lamp biomicroscopy, dilated fundus examination, and fluorescein angiography (FFA) to evaluate posterior segment inflammation and vasculitis. Optical coherence tomography angiography (OCTA) and ocular ultrasonography were performed as auxiliary investigations when indicated. Best-corrected visual acuity (BCVA), intraocular pressure (IOP), and detailed findings from anterior and posterior segment examinations were recorded.

### 2.4 Treatment protocol

Anterior segment inflammation was managed with topical corticosteroids and mydriatic agents. Posterior segment involvement was treated with systemic corticosteroids, typically combined with immunosuppressive agents (e.g., cyclosporine, azathioprine). Biological agents (e.g., interferon-α, adalimumab) were considered in cases refractory to conventional therapy. Elevated IOP was managed with anti-glaucoma medications, laser treatment, or surgery as needed.

### 2.5 Systemic disease classification

BD was classified as severe or non-severe based on a clinical severity scoring system, and as complete or incomplete based on the presence of major systemic manifestations, as described in previous studies (17–20).

### 2.6 Statistical analysis

Statistical analysis was performed using SPSS version 31.0 and R version 4.5.2. Kaplan–Meier analysis was used to estimate cumulative visual loss. Chi-square tests and mixed-effects logistic regression models (accounting for inter-eye correlation) were employed to identify factors associated with visual outcomes. A two-sided p-value < 0.05 was considered statistically significant.

## 3 Results

A total of 153 patients with Behçet’s uveitis (BU), involving 268 eyes, were included in this study. Among them, 119 (77.8%) were male and 34 (22.2%) were female. The median follow-up duration was 30 months. Bilateral involvement was observed in 129 patients (84.3%). All patients received systemic glucocorticoid therapy, among whom 19 had discontinued glucocorticoids by the final follow-up. Immunosuppressive agents were administered to all patients, with 18 discontinuing treatment by the end of follow-up. During the follow-up period, 28 patients were treated with biologic agents, of whom 15 had discontinued biologic therapy at the last follow-up (**Table 1**).

**Table 1:** Ongoing and previous treatments in patients with Behçet’s uveitis.

Treatment category	Patients, n(%)
Systemic corticosteroids	
Previous use	153 (100%)
Ongoing use at last visit	134 (87.6%)
Conventional immunosuppressants	
Previous use	153 (100%)
Ongoing use at last visit	135 (88.2%)
Biologic agents	
Previous use	28 (18.3%)
Ongoing use at last visit	13 (8.5%)

**Figure 1:**
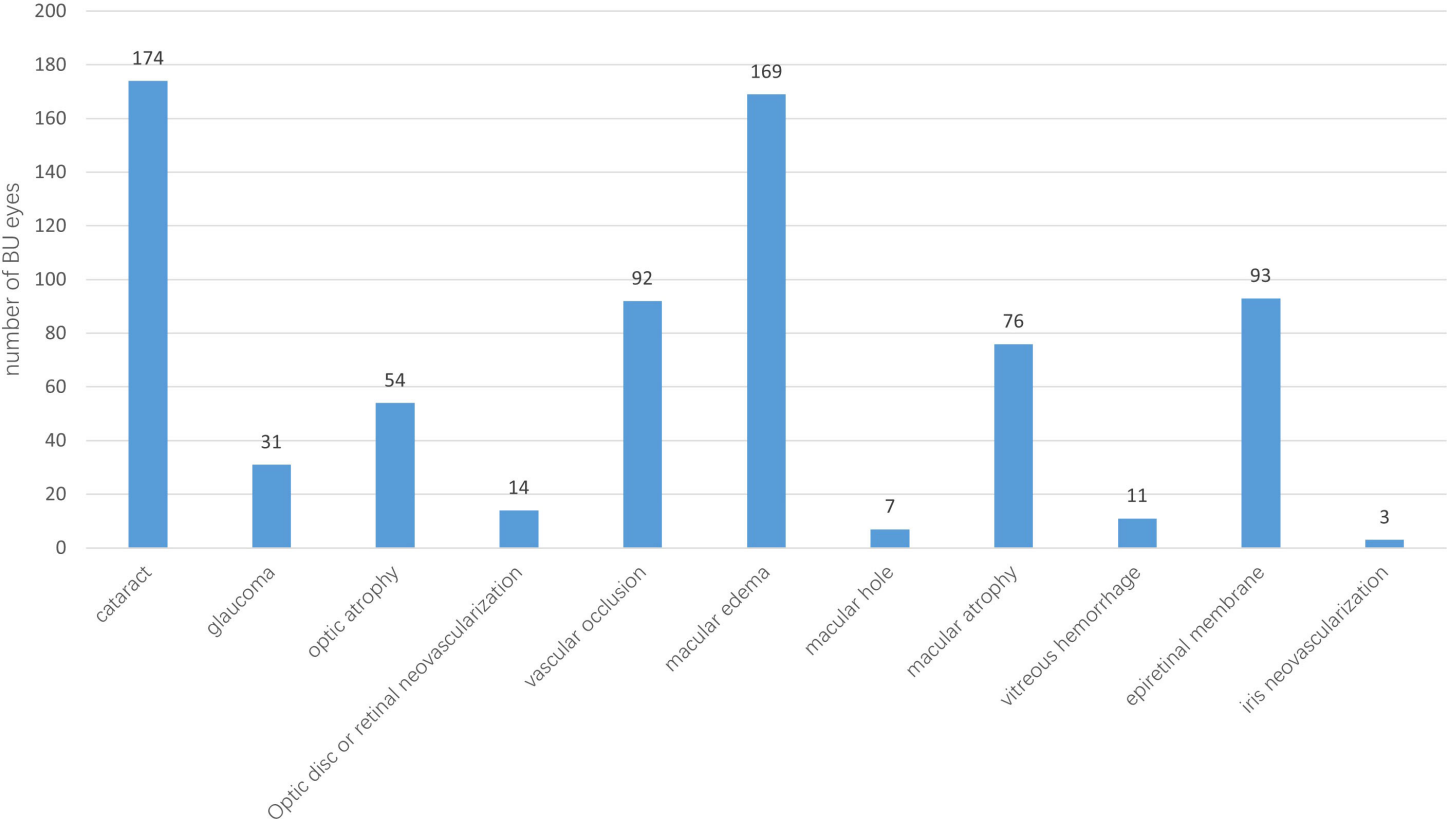
Ocular complications of patients with Behcet’s uveitis (BU).

**Figure 2:**
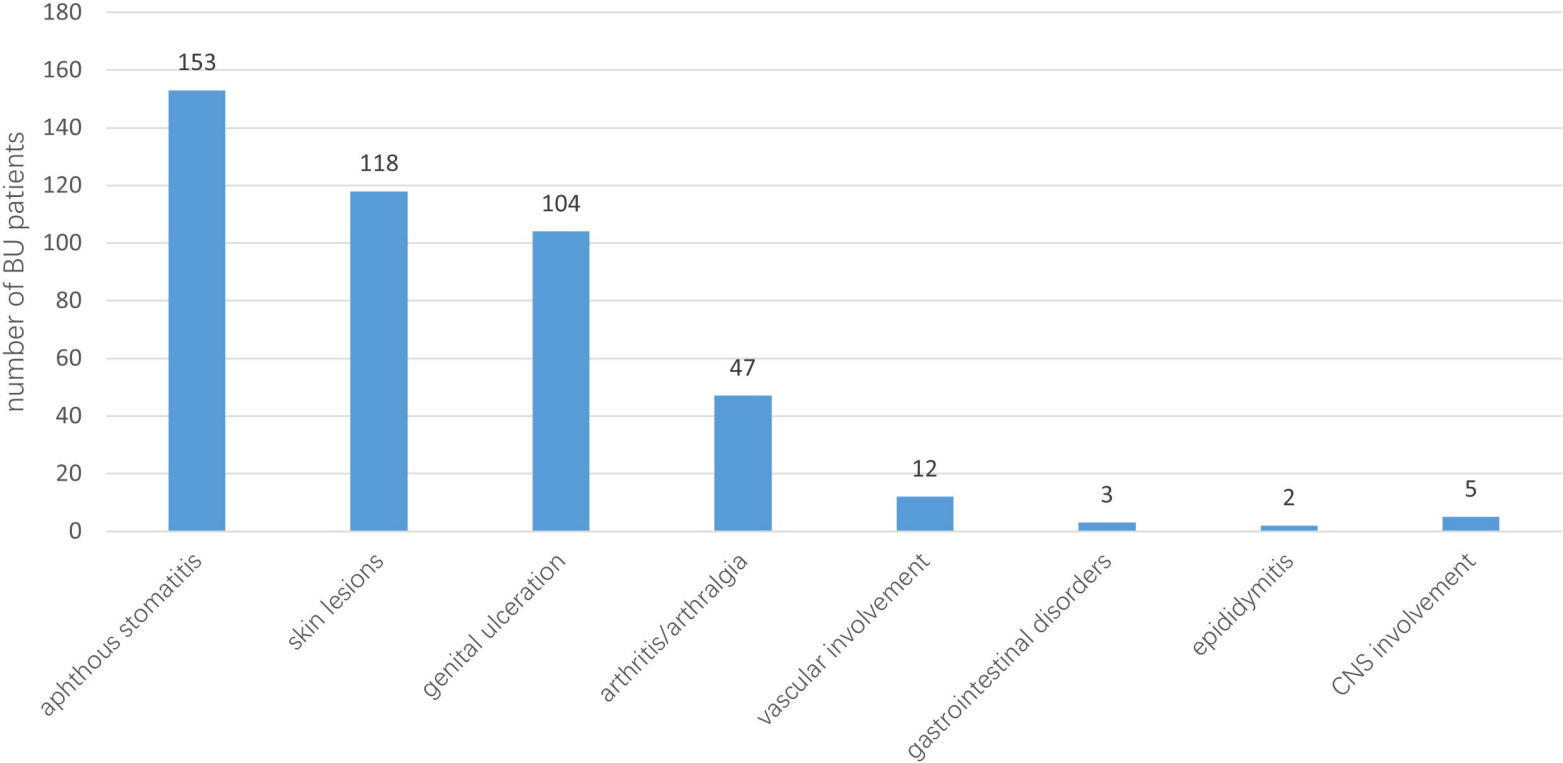
Extraocular manifestations of patients with Behcet’s uveitis (BU).

Panuveitis was the most common type of uveitis, affecting 242 eyes (90.3%), followed by posterior uveitis, observed in 21 eyes (7.8%). Cataract was the most prevalent ocular complication, present in 174 eyes (64.9%), macular edema was the second most common complication, occurring in 169 eyes (63.1%). During the follow-up period, glaucoma was diagnosed in 31 eyes (11.6%). After receiving medical, laser, or surgical treatments (alone or in combination), all eyes achieved satisfactory intraocular pressure control. Detailed data on ocular complications are shown in **Figure 1**. Recurrent aphthous stomatitis was the most common extraocular manifestation, occurring in all 153 patients. Skin lesions followed in frequency, affecting 118 patients (77.1%). Detailed data on extraocular manifestations are provided in **Figure 2**.

**Figure 3:**
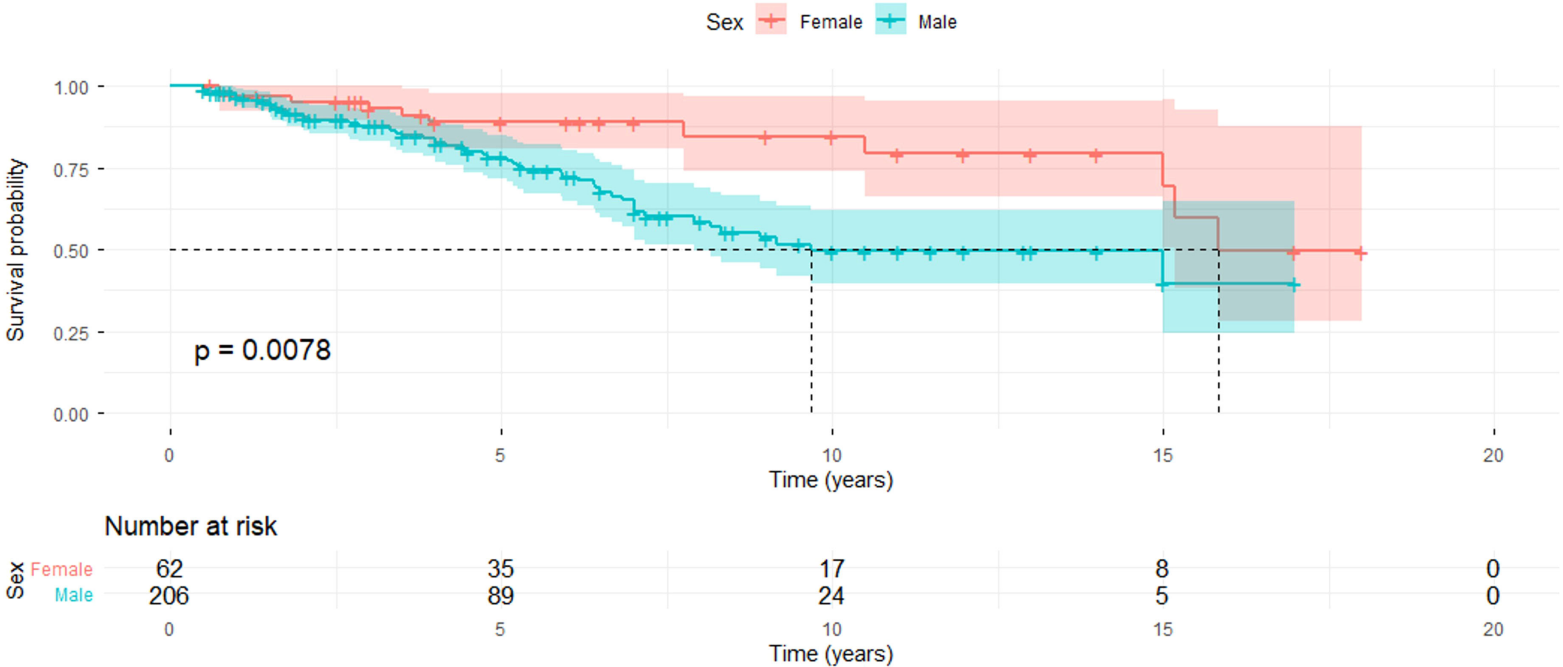
Kaplan-Meier curves depicting the cumulative survival of eyes with a potential visual acuity worse than 20/200 in patients with behcet’s uveitis (BU).

At the initial visit, best-corrected visual acuity (BCVA) was worse than 20/200 in 63 of 268 eyes (23.5%) and worse than 20/400 in 48 eyes (17.9%). At the final follow-up, 68 eyes (25.4%) had BCVA worse than 20/200, and 52 eyes (19.4%) had BCVA worse than 20/400, with a median follow-up duration of 30 months. Kaplan–Meier survival analysis demonstrated that the cumulative risk of loss of useful vision (BCVA <20/200) was 3.5% at 1 year, 11.0% at 2 years, 19.2% at 5 years, and 41.4% at 10 years. Notably, male patients experienced visual impairment significantly earlier than female patients (**Figure 3**).

In group comparisons, there were significant differences in the incidences of retinal vascular occlusion (p<0.001), glaucoma (p<0.001), macular damage (p<0.001), optic nerve atrophy (p<0.001), complicated cataract (p<0.001), and complete BD (p=0.021) between patients with different visual outcomes. However, the severity of systemic BD (p=0.306) did not differ significantly between the two groups (**Table 2**).

**Table 2:** Comparisons of ocular and systemic features between eyes with different visual outcome.

Clinical feature	Good visual outcome n=200	Poor visual outcome n=68	p value
Retinal vascular occlusion	48(24%)	44(64.7%)	<0.001
Glaucoma	15(7.5%)	16(23.5%)	<0.001
Macular damage	126(63%)	64(94.1%)	<0.001
Optic nerve atrophy	17(8.5%)	37(54.4%)	<0.001
Complicated cataract	113(56.5%)	61(89.7%)	<0.001
Severe systemic BD	50(25%)	22(32.4%)	0.306
Complete BD	101(50.5%)	46(67.6%)	0.021

Poor visual outcome was defined as best-corrected visual acuity worse than 20/200 at the last follow-up. BD, Behçet’s disease. Eyes were classified as “Good” or “Poor” based on final visual acuity. The inter-group comparison is based on individual eyes.

To account for inter-eye correlation in patients with bilateral involvement, a mixed-effects logistic regression model was employed, with patient ID specified as a random effect. Age, sex, disease duration, RVO, glaucoma, macular damage, optic nerve atrophy, complicated cataract, severe BD, complete BD and the use of biological agents were included as fixed effects in the analysis of factors associated with visual outcomes. Macular damage (p=0.027, OR = 3.70) and optic nerve atrophy (p<0.001, OR = 6.47) were significantly associated with visual outcomes in patients with BU, while complete BD (p=0.072, OR = 2.00) showed a trend toward association (**Table 3**).

**Table 3:** Mixed-effects logistic regression analysis evaluating the associations between potential risk factors and the visual outcome of BU eyes.

Variables	Category	Univariate OR (95% CI)	P value	Multivariate OR (95% CI)	P value
Age (years)	/	1.01 (0.98, 1.04)	0.551	0.99 (0.95, 1.03)	0.603
	/	Ref		Ref	
Gender	Male	1.79 (0.86, 3.75)	0.123	1.30 (0.524, 3.23)	0.572
	Female	Ref		Ref	
Disease duration (years)	/	1.10 (1.03, 1.18)	0.004	0.995 (0.91, 1.09)	0.907
	/	Ref		Ref	
Retinal vascular occlusion	Yes	5.81 (3.21, 10.50)	<0.001	1.27 (0.55, 2.93)	0.569
	No	Ref		Ref	
Glaucoma	Yes	3.87 (1.68, 8.93)	0.001	2.21 (0.81, 6.02)	0.121
	No	Ref		Ref	
Macular damage	Yes	9.40 (3.21, 27.50)	<0.001	3.70 (1.16, 11.80)	0.027
	No	Ref		Ref	
Optic nerve atrophy	Yes	12.85 (6.45, 25.60)	<0.001	6.47 (2.52, 16.70)	<0.001
	No	Ref		Ref	
Complicated cataract	Yes	6.71 (2.93, 15.39)	<0.001	2.20 (0.85, 5.68)	0.104
	No	Ref		Ref	
Severe systemic BD	Yes	1.45 (0.78, 2.70)	0.245	1.35 (0.60, 3.00)	0.467
	No	Ref		Ref	
Complete BD	Yes	2.06 (1.14, 3.73)	0.017	2.00 (0.94, 4.25)	0.072
	No	Ref		Ref	
Biological agents	Yes	1.04 (0.512, 2.10)	0.921	0.89 (0.37, 2.12)	0.780
	No	Ref		Ref	

Marginal R²=0.416. The outcome is whether the visual acuity at the last follow-up is less than 20/200. Parameters are binarized by median, and P values < 0.05 are considered to be statistically significant. Macular damage include macular edema, epi-retinal membrane, macular atrophy and macular hole. The mixed-effects logistic regression analysis was based on individual eyes.

## 4 Discussion

In this retrospective cohort study, we evaluated the visual prognosis of Behçet’s uveitis (BU) in a Chinese population and explored factors associated with poor visual outcomes. Our findings indicate that, despite the widespread use of contemporary systemic immunosuppressive and biologic therapies, visual impairment remains common among patients with BU, with more than one-quarter experiencing severe visual loss during the disease course. Cataract and macular edema were the most frequent ocular complications, whereas macular damage and optic nerve atrophy emerged as the predictors of poor visual prognosis. Notably, systemic disease severity was not significantly associated with visual outcomes, while complete-type Behçet’s disease demonstrated a borderline association.

Although macular damage and optic nerve atrophy remained dominant prognostic factors, the overall proportion of eyes progressing to severe visual impairment in the present study was comparable to or lower than that reported in earlier series (21–23). This apparent improvement in visual outcomes may be attributable, at least in part, to the increasing use of biologic agents in routine clinical practice in recent years (24, 25).

However, biologic therapy was not identified as an independent protective factor against severe visual impairment in the regression analyses, and this finding should be interpreted with caution. In our cohort, biologic agents were preferentially prescribed to patients with more severe or refractory disease who had failed conventional immunosuppressive therapy. Such confounding by indication may have attenuated the observable therapeutic benefit in multivariable models. Importantly, despite unfavorable baseline characteristics, overall visual outcomes in our cohort appeared improved compared with those reported in earlier studies, suggesting that biologic therapy may still contribute to better visual prognosis at the population level (21, 26).

The lack of a significant association between systemic disease severity and visual outcomes may reflect the inherent heterogeneity of Behçet’s disease (27, 28). Previous studies have shown that patients with intestinal Behçet’s syndrome rarely develop ocular involvement, whereas those with ocular Behçet’s syndrome often show few abnormalities in systemic inflammation, including inflammatory markers and involvement of other organs (8, 29). Consistent with our findings, these observations suggest that visual outcomes in ocular Behçet’s syndrome are primarily determined by intraocular inflammatory activity, the presence of ocular complications, timing of treatment initiation, and individual drug responses, rather than by the overall activity or severity of systemic disease. Accordingly, systemic disease severity may not directly predict ocular prognosis, highlighting the importance of independent evaluation and tailored management strategies for ocular Behçet’s syndrome in clinical practice.

In contrast, complete-type Behçet’s disease represent a distinct disease phenotype characterized by more extensive immune dysregulation and a higher propensity for posterior segment involvement, which could partially explain its trend toward poorer visual outcomes. Moreover, with the widespread use of aggressive immunosuppressive and biologic therapies, the historically reported association between systemic disease severity and ocular prognosis may have been attenuated in contemporary cohorts (30).

Our study has several limitations. First, as a retrospective *post hoc* analysis, standardized imaging follow-up intervals were unavailable, which may have resulted in underestimation of mild macular or retinal vascular changes. Second, treatment response was not systematically evaluated. Third, all patients were recruited from a single tertiary referral center, which may have introduced selection bias. In addition, although validated scoring systems such as the Behçet’s Disease Ocular Attack Score 24 (BOS24) are useful for quantifying acute ocular inflammatory activity, their application in the present study was limited by incomplete standardized documentation across visits and by the study’s focus on long-term visual outcomes rather than attack-based disease activity.

## 5 Conclusion

In this retrospective cohort study of a Chinese population with Behçet’s uveitis, visual impairment remained common despite the widespread use of modern immunosuppressive and biologic therapies. Long-term visual prognosis was primarily determined by irreversible ocular structural damage, particularly macular damage and optic nerve atrophy, whereas systemic disease severity showed limited predictive value. Although overall visual outcomes appeared improved compared with earlier reports, these findings underscore the importance of early recognition and effective control of posterior segment inflammation to prevent permanent visual loss in patients with Behçet’s uveitis.

## Data Availability

The raw data supporting the conclusions of this article will be made available by the authors, without undue reservation.
